# Chemotactic Motility of *Pseudomonas fluorescens* F113 under Aerobic and Denitrification Conditions

**DOI:** 10.1371/journal.pone.0132242

**Published:** 2015-07-10

**Authors:** Candela Muriel, Blanca Jalvo, Miguel Redondo-Nieto, Rafael Rivilla, Marta Martín

**Affiliations:** Departamento de Biología, Universidad Autónoma de Madrid, Madrid, Spain; University of the West of England, UNITED KINGDOM

## Abstract

The sequence of the genome of *Pseudomonas fluorescens* F113 has shown the presence of multiple traits relevant for rhizosphere colonization and plant growth promotion. Among these traits are denitrification and chemotactic motility. Besides aerobic growth, F113 is able to grow anaerobically using nitrate and nitrite as final electron acceptors. F113 is able to perform swimming motility under aerobic conditions and under anaerobic conditions when nitrate is used as the electron acceptor. However, nitrite can not support swimming motility. Regulation of swimming motility is similar under aerobic and anaerobic conditions, since mutants that are hypermotile under aerobic conditions, such as *gacS*, *sadB*, *kinB*, *algU* and *wspR*, are also hypermotile under anaerobic conditions. However, chemotactic behavior is different under aerobic and denitrification conditions. Unlike most pseudomonads, the F113 genome encode three complete chemotaxis systems, Che1, Che2 and Che3. Mutations in each of the *cheA* genes of the three Che systems has shown that the three systems are functional and independent. Mutation of the *cheA1* gene completely abolished swimming motility both under aerobic and denitrification conditions. Mutation of the *cheA2* gene, showed only a decrease in swimming motility under both conditions, indicating that this system is not essential for chemotactic motility but is necessary for optimal motility. Mutation of the *cheA3* gene abolished motility under denitrification conditions but only produced a decrease in motility under aerobic conditions. The three Che systems proved to be implicated in competitive rhizosphere colonization, being the *cheA1* mutant the most affected.

## Introduction

The *Pseudomonas fluorescens* complex comprises a group of related pseudomonads that are often found saprophytically associated with plants. A large number of strains belonging to this group have been found to colonize the rhizosphere and the endophytic compartments and to positively influence plant growth, either directly through biofertilization or manipulation of the plant hormone balance, or indirectly through biological control of pathogens [1]. A series of recent phylogenomic studies [2–4] have shown that the *P*. *fluorescens* complex contains up to five different phylogenetic subgroups and that many traits putatively responsible of the plant growth promoting abilities are phylogenetically distributed.

*P*. *fluorescens* F113 belongs to Subgroup I and is phylogenetically related to strains that have been classified as *P*. *brassicacearum*. F113 is a plant growth promoting rhizobacteria (PGPR) that is able to colonize the rhizosphere [5], influencing plant growth directly, by phosphate mobilization [6] and degradation of the plant hormone ethylene [4], and indirectly by the production of siderophores, fungicides (DAPG) and competition for niche with plant pathogens [7]. F113 can colonize the rhizosphere of a wide variety of plants and has been used as a model for rhizosphere colonization [8–13]. The full genomic sequence of F113 is available and analysis of the genome has shown the presence of numerous traits that are likely to be involved in its rhizosphere colonization and PGPR abilities [4].

Among these traits is denitrification. The F113 genome contains the *nar*, *nir*, *nor* (two sets) and *nos* (two sets) gene clusters, which encode the enzymes required for nitrate, nitrite, nitric oxide and nitrous oxide reduction, respectively [4]. The presence of these sets of genes indicates that F113 is likely to oxidize nitrate to dinitrogen. The ability to use nitrate and nitrite under anaerobic conditions as the final electron acceptors has been shown for F113 [4]. Denitrification has been observed in several pseudomonads species such as *P*. *aeruginosa* and *P*. *stutzeri* [14]. However, although denitrification occurs within the *P*. *fluorescens* complex [15, 16], it seems to be restricted to a limited number of strains which harbor denitrification genes [4]. Denitrification genes are present in all Subgroup I sequenced strains. It has been shown that there is a positive correlation between denitrification ability and rhizosphere colonization [17, 18].

Another important trait for rhizosphere colonization is chemotactic motility. In several pseudomonads strains, mutants affected either in flagellar genes [19, 20] or chemotaxis genes [21] are severely impaired in the competitive colonization of the rhizosphere. In F113, motility is one of the most important traits for rhizosphere colonization since hypermotile mutants affected in regulatory genes show enhanced competitive colonization ability [5] and are selected in the rhizosphere environment [11]. Among these mutations are those affecting flagella synthesis such as mutants in the Gac system, in *algU*, *kinB*, *sadB* and in *amrZ* [7, 22, 23, 24] or affecting flagella rotation such as mutants in *wspR* and in *bifA* [25].

The chemotactic apparatus of pseudomonads is encoded by the *che* genes [26, 27]. It consists in a phosphorely in which phosphorylation of CheY by CheA results in the interaction of CheY with the flagellar rotor and changes in flagella rotation. Environmental signaling to CheA is provided by membrane-bound or cytoplasmic methyl acceptor proteins (MCPs). Other proteins such as CheB, CheD, CheV, CheW, CheR and CheZ also participate in the signal transduction process and in resetting the system [28]. Most pseudomonads strains possess one or two sets of the chemotaxis genes [4, 29]. However, F113 and *P*. *brassicacearum* strains such as NFM421 possess three sets of the *che* genes that might conform three distinct and independent chemotaxis apparatus [4].

The aim of this work was to investigate motility regulation in *P*. *fluorescens* F113 under anaerobic denitrification conditions and to determine the functionality of the three chemotactic apparatus and their roles under aerobic and anaerobic conditions. The implication of each of the chemotactic apparatus in competitive rhizosphere colonization was also investigated.

## Results

### Growth and motility of *P*. *fluorescens* F113 under denitrification conditions

**Fig 1:**
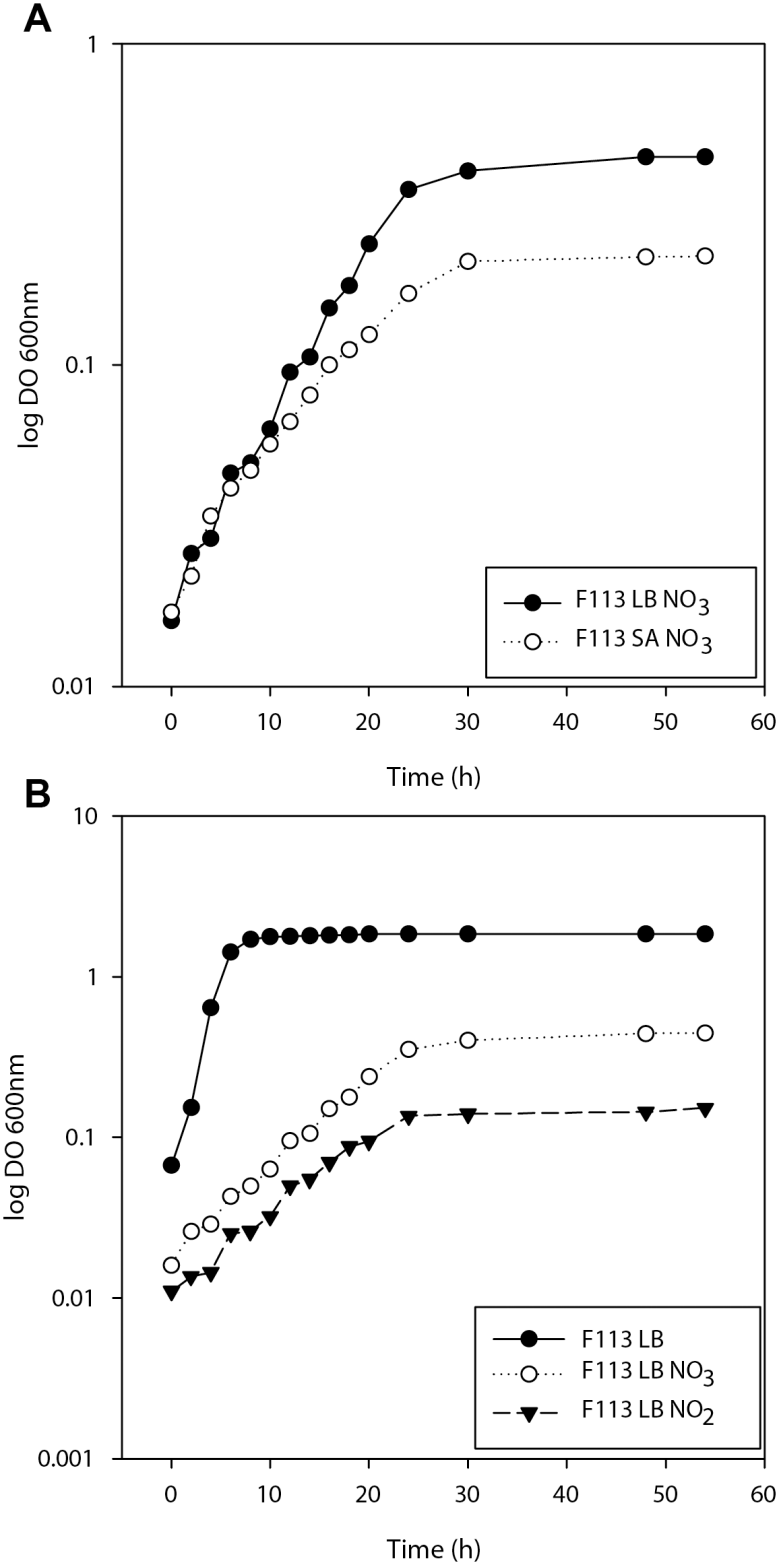
Growth of *P*. *fluorescens* F113 under denitrification conditions. Growth curves of F113 with different final electron acceptors. Nitrate concentration was 40mM, nitrite concentration was 10mM. Anaerobic conditions were obtained by flushing inoculated medium with argon. For anaerobic conditions, air-tight tubes were used. F113 was unable to grow anaerobically in LB or SA media without nitrate or nitrite. Experiments were done in triplicate (A) Growth curves of F113 in LB and SA media supplemented with nitrate (B) Growth curves of F113 in LB medium (aerobic), LB supplemented with nitrate and LB supplemented with nitrite.

Growth of F113 was tested using nitrate and nitrite as final electron acceptors under anaerobic conditions in two different media: LB and SA. In order to estimate optimal concentration of electron acceptors, both media were supplemented with increasing concentrations of nitrate and nitrite. Under anaerobic conditions F113 was unable to grow in the absence of nitrate or nitrite in both media. 40 mM nitrate and 10 mM nitrite yielded maximum cell density in both media (Table 1). These concentrations were used for subsequent growth experiments. It is interesting to note that opposed to growth under aerobic conditions, the siderophore pyoverdine was not produced in the iron limited medium SA under denitrification conditions. As shown in Fig 1A, growth rate and yield on nitrate were higher in LB medium than in SA medium. On nitrite, growth was very poor in SA medium. Therefore, LB was used for subsequent growth experiments. Fig 1B shows the growth of F113 using either of the three electron acceptors. Growth under aerobic conditions showed higher growth rate and yield. Under anaerobic conditions, no differences were observed on the growth rate when using nitrate or nitrite. However, yield was higher when nitrate was the final electron acceptor.

**Table 1:** Yield (max OD_600_) of *Pseudomonas fluorescens* F113 grown under anaerobic conditions in the presence of different concentrations of final electron acceptors (nitrate and nitrite) in SA and LB media.

Growth with NO_3_^-^ as electron acceptor			Growth with NO_2_^-^ as electron acceptor		
KNO_3_	SA	LB	NaNO_2_	SA	LB
0	<0.01	<0.03	0	<0.01	<0.03
20 mM	0.055	0.243	5 mM	0.030	0.138
40 mM	0.095	0.733	10 mM	0.074	0.388
60 mM	0.070	0.654	15 mM	0.042	0.219
80 mM	0.062	0.448	20 mM	0.049	0.316
100 mM	0.061	0.300	25 mM	0.035	0.305

We also tested swimming motility under denitrification conditions. It was observed that F113 was able to move in SA and LB plates supplemented with nitrate but was non-motile with nitrite. Motility was higher in SA medium than in LB medium. Therefore SA was used in subsequent motility experiments. When F113 swims under nitrate respiring conditions no pyoverdine production was observed in SA plates, conversely to swimming in this medium under aerobic conditions. In order to test whether the lack of motility under nitrite respiring conditions were due to lack of flagella production, cells grown in SA + nitrite were observed by electron and optic microscopy and they showed normal flagellation (S1 Fig). Furthermore, when these cells were transferred to an aerobic atmosphere, motility was restored immediately, as judged by phase-contrast microscopy, indicating that the lack of motility was probably due to shortage of energy under nitrite conditions.

### Motility regulation under denitrification conditions

**Fig 2:**
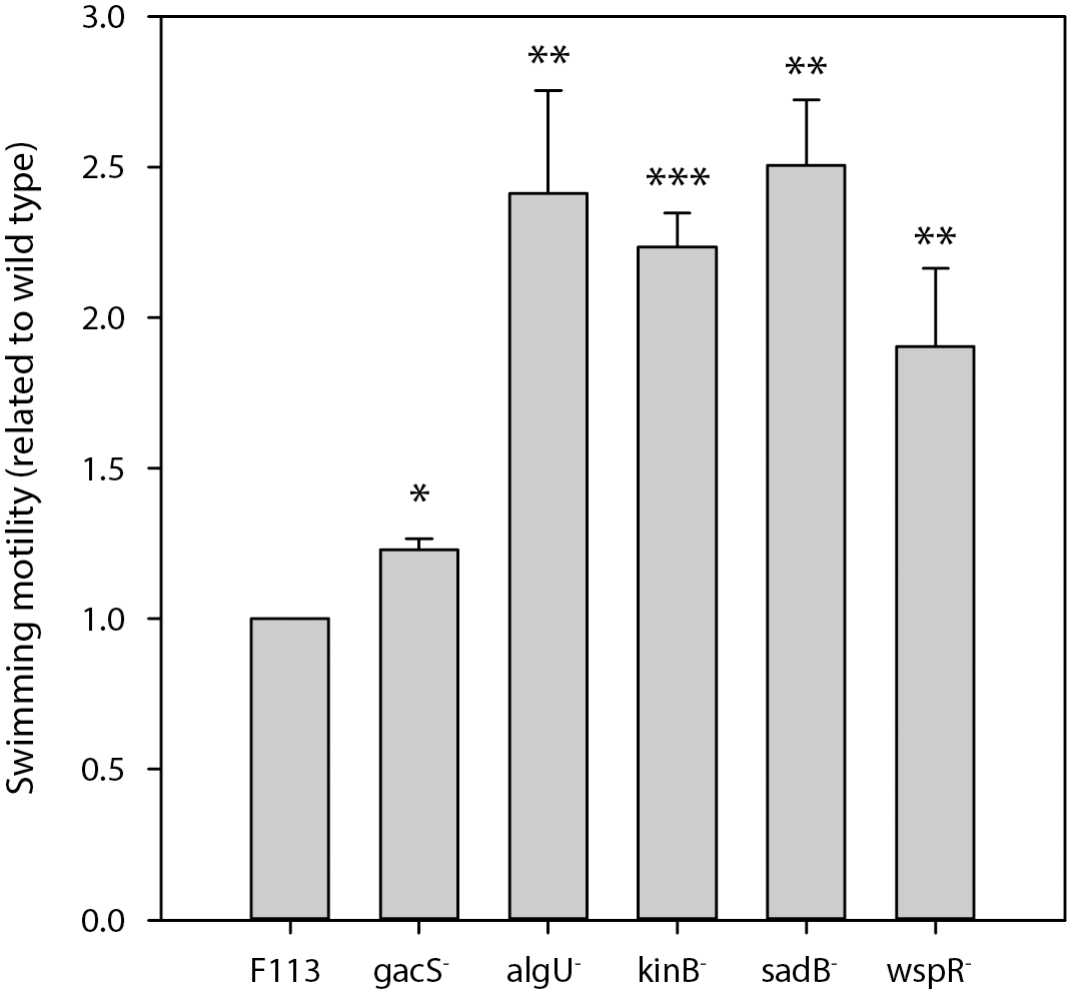
Motility regulation under denitrification conditions. Swimming motility phenotype of *P*. *fluorescens* F113 and isogenic mutants affected in swimming motility. Experiments were done on SA (0.3% agar) under anaerobic (denitrification) conditions. Haloes were measured 48 h after inoculation. Experiments were performed three times in triplicate. Statistically significant results are shown as: * (p<0.1); ** (p<0.05); *** (p<0.01).

Under aerobic conditions, swimming motility is regulated at the levels of flagella synthesis and flagella rotation [22, 24]. In order to test whether this regulation also exists under denitrification conditions, the swimming motility of several hypermotile mutants affected in either pathways was tested in SA + nitrate plates under anaerobic conditions. As shown in Fig 2, the *gacS*, *sadB*, *kinB* and *algU* mutants showed a swimming behavior similar to their hypermotile phenotype under aerobic conditions. These mutants are affected in flagella synthesis. Similarily, a *wspR* mutant that is hypermotile under aerobic conditions, is also hypermotile under anaerobic, denitrification conditions. The *wspR* gene encodes a diguanylate cyclase probably involved in the regulation of flagella rotation and is not implicated in the regulation of flagella synthesis [24]. These results indicates that swimming motility regulation is very similar, if not identical under aerobic and denitrification conditions.

### *P*. *fluorescens* F113 possesses three independent and functional chemotactic systems

**Fig 3:**
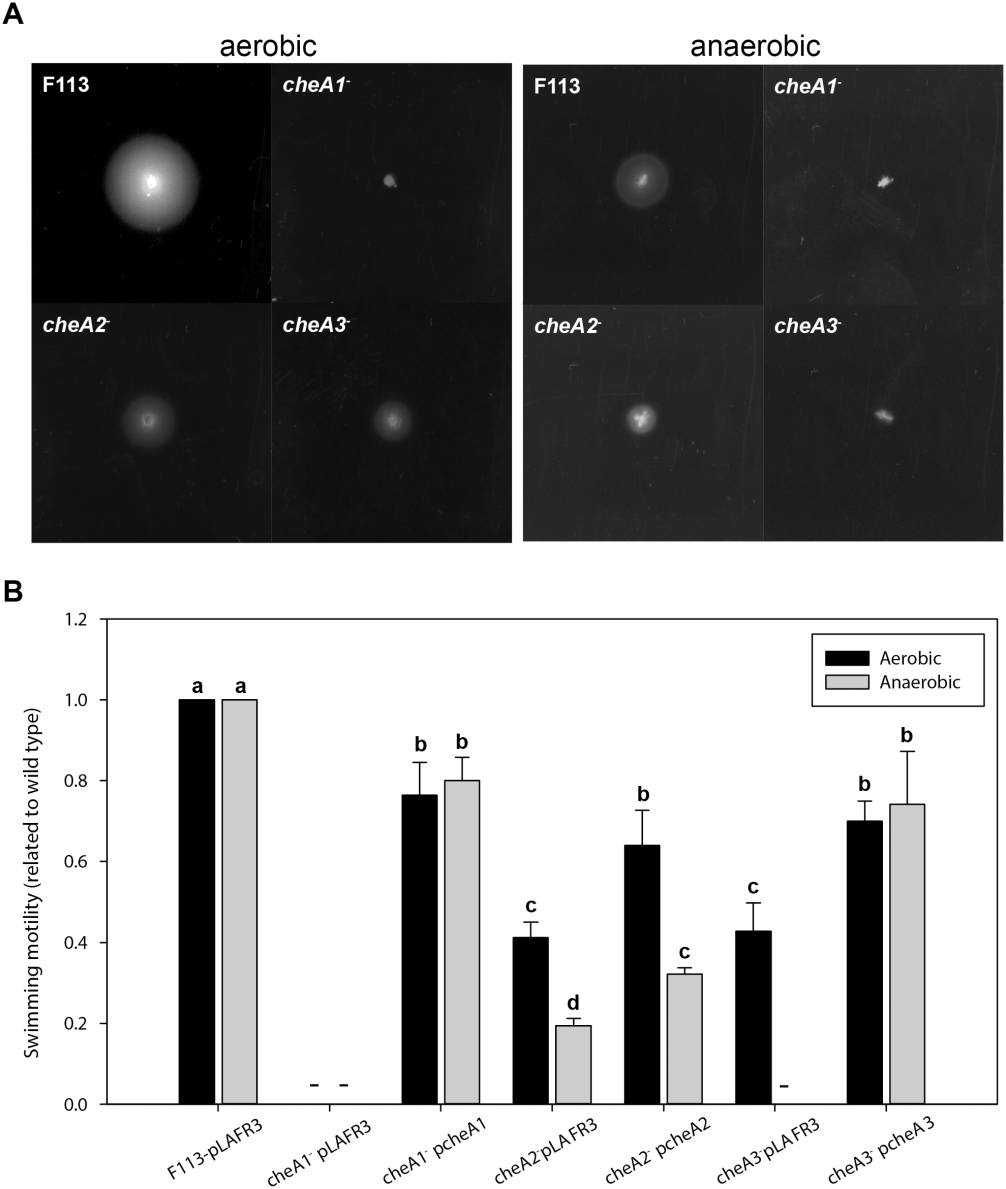
*P*. *fluorescens* F113 possesses three independent and functional chemotactic systems. (A) Swimming motility phenotype of *P*. *fluorescens* F113 and its mutants affected in either of the three *cheA* genes under aerobic and anaerobic (denitrification) conditions. Swimming haloes were measured after 18 h (aerobic) and 48h (anaerobic) inoculation on SA (0.3% agar). The experiments were repeated three times in triplicate. Typical images are shown. (B) Complementation analysis of the three *cheA* mutants. Each of the mutants was complemented with cosmids from the F113 gene library which hybridized with each of the *cheA* probes: pcheA1 (pBG2076); pcheA2 (pBG1994); pcheA3 (pBG1989). The pLAFR3 vector was introduced in F113 and each of the three mutants for controls. Swimming motility haloes were determined as above on medium supplemented with tetracycline. The experiments were repeated three times in triplicate. (–) indicates not detectable movement. Different letters indicate statistically significant differences (p<0.05).

The genome sequence of F113 showed that it contains three full chemotaxis systems (Che1, Che2 and Che3), plus thirty-six putative chemoreceptors (MCPs). The genetic organization of these gene clusters is shown in S2 Fig. In order to test the functionality of these systems, mutants affecting each of the three CheA genes were constructed. The three mutants were tested for growth in SA and LB media under aerobic and denitrification conditions. None of the mutants showed differences in growth rate or yield in any of the media or growth conditions. The mutants were also observed by phase contrast microscopy. Although the three mutants were motile, they showed important differences with the wild-type strain. While F113 cells showed a swimming pattern characterized by frequent changes of direction, *cheA1* ^-^ and *cheA3* ^-^ cells did not change direction and only followed straight trajectories. An intermediate phenotype was observed for *cheA2* ^-^ cells, where only a few cells changed direction occasionally. Fig 3 shows the swimming phenotypes of each of the mutants under aerobic and anaerobic conditions. Under aerobiosis, the major effect was observed in the *cheA1* mutant that was unable to swim under these conditions. Mutation of *cheA2* and *cheA3* also resulted in a reduction in motility, as judged by halo reduction, an effect that was higher in the *cheA3* mutant. As shown in Fig 3B cosmids from an F113 genomic library which contained the genes encoding the respective Che systems were isolated by colony hybridization and were able to partially complement the swimming defects of the mutants. These results show that the three Che systems are functional and independent in F113. The swimming motility of the three mutants was also tested under denitrification conditions. As shown in Fig 3 the three mutants were also affected in swimming motility under anaerobic conditions. The phenotypes of *cheA1* and *cheA2* were similar to their phenotypes under aerobic conditions. However, under anaerobic denitrification conditions, the *cheA3* mutant was non-motile, presenting the same phenotype that the *cheA1* mutant. The *cheA1*, *cheA2* and *cheA3* phenotypes were partialy complemented by the cosmids containing their wild-type counterparts (Fig 3B). These results indicate that chemotaxis is different under aerobic and denitrification conditions. They also show that *cheA1* is essential for chemotactic motility both under aerobic and denitrification conditions, while *cheA3* is essential for chemotactic motility only under denitrification conditions.

### The three chemotaxis systems are important for rhizosphere colonization

**Fig 4:**
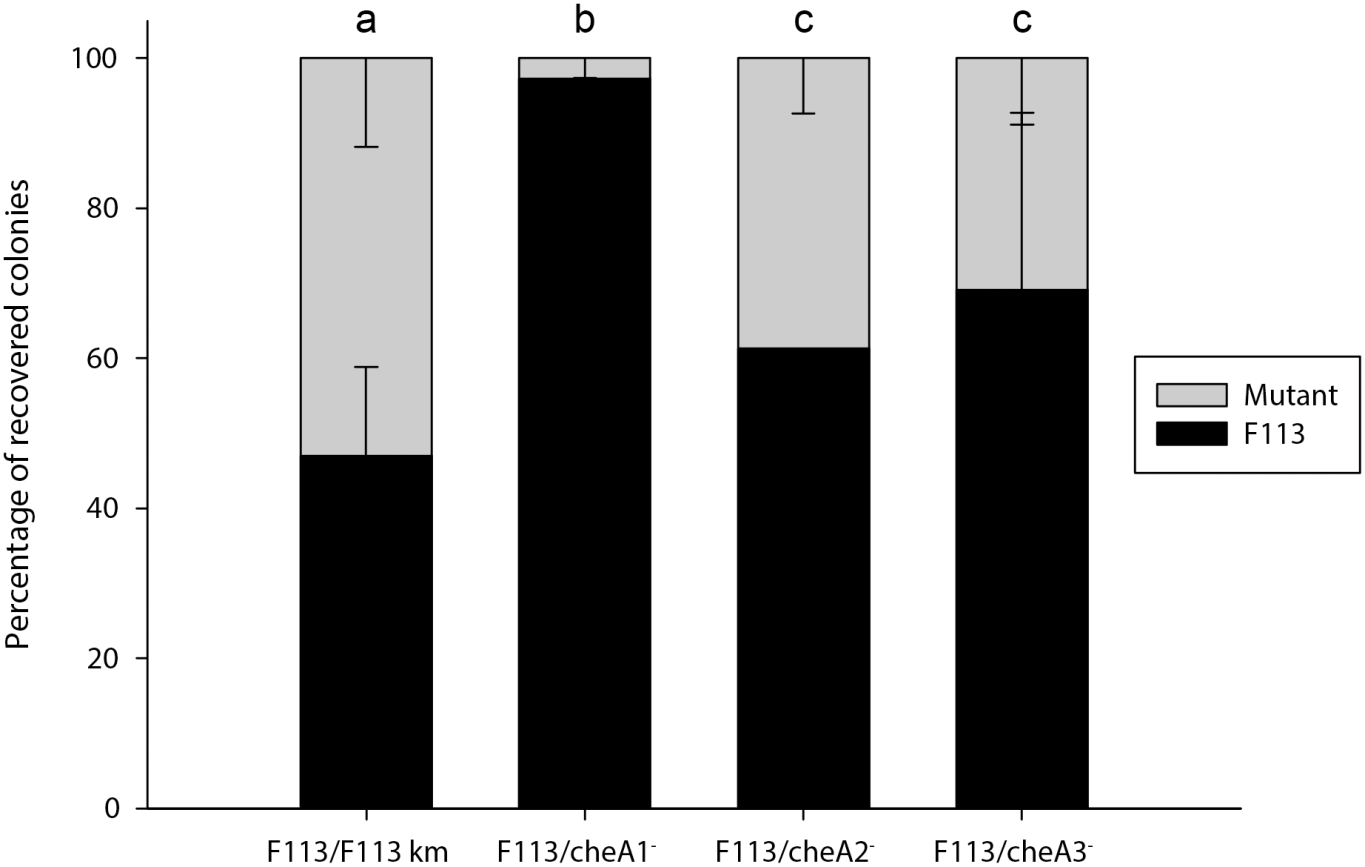
The three chemotaxis systems are important for rhizosphere colonization. Competitive colonization root-tip assay of *P*. *fluorescens* F113 and isogenic mutants affected in either of the *cheA* genes. Equal amounts (10^8^ cells) of F113 and its competitors were inoculated per plant. F113 was also compared with an isogenic strain tagged with a kanamycin resistance gene as a control. Experiments were done in triplicate with 20 plants in each experiment. Different letters indicate statistically significant differences (p<0.05).

Chemotactic motility is one of the most important traits for competitive rhizosphere colonization. In order to investigate the relative importance of each of the Che systems for colonization, we tested the performance of each of the *cheA* mutants in competition with the wild-type strain, with a root-tip assay. As shown in Fig 4, the three mutants were impaired in competitive colonization of the root tip, showing significant difference (p< 0.05) when competing with the wild-type strain. The higher defect was observed with the *cheA1* mutant that was displaced by the wild-type strain. The *cheA2* and *cheA3* mutants were always recovered in lower numbers than the wild-type strain, therefore showing a minor but significant impairment in competitive rhizosphere colonization. No significant difference in competitivity was observed between the *cheA2* and *cheA3* mutants. The three chemotaxis systems are therefore required for optimal performance in the rhizosphere.

## Discussion

*Pseudomonas fluorescens* F113 can grow both aerobically and anaerobically. Anaerobic growth is based in denitrification, being able to use both nitrate and nitrite as final electron acceptors. The presence in the F113 genome of complete sets of *nor* and *nos* genes [4], suggests that nitric oxide and nitrous oxide are also putative electron acceptors for this strain and that molecular dinitrogen is the final product of denitrification. Under anaerobic conditions, growth yield for F113 is higher when nitrate is used as the electron acceptor and when the bacterium is grown on a rich medium. The results presented in Table 1 show that increasing the concentration of the electron acceptors, yield also increased, indicating that in our experimental conditions, growth is limited by the electron acceptor. However levels of nitrate higher than 40 mM or nitrite 10 mM reduced yield, indicating toxicity of both nitrogenated compounds. Although these concentrations are much higher than those encountered in soils, it is likely that growth on nitrate and nitrite are physiological relevant, since for *P*. *aeruginosa* optimal growth has been shown to occur at 100 mM nitrate [30], but growth has been observed with nitrate concentrations as little as 62.5 μM [31]. When denitrification occurs at optimal electron acceptors concentration (Fig 1), the growth rate does not appear to depend on the availability of other nutrients, including iron. It is therefore likely that in the rhizosphere, where nitrate and nitrite will be limiting, growth rates are lower than those observed here. The importance of denitrification has been highlighted in a recent study of 23 strains of pseudomonads able to colonize the tomato rhizosphere under natural soil conditions [18]. In this study, it was shown that the ability to use alternative electron acceptors was more important for rhizosphere competence than the ability to use specific carbon sources. Furthermore, survival in the rhizosphere was also related to denitrification ability.

Under anaerobic conditions, *P*. *fluorescens* F113 is motile when nitrate is the final electron acceptor, but not when nitrite is used. However, cells grown on nitrite are flagellated, as shown by microscopy inspection and by its immediate motility after shifting to aerobic conditions. It is likely that the shortage in energy provided by nitrite reduction is responsible of the lack of motility under these conditions. Swimming motility haloes are smaller under anaerobic conditions than under aerobic conditions, suggesting that reduced motility is also related with the amount of energy harvested by the different electron acceptors. Regarding motility regulation, it seems to be very similar, if not identical under aerobic and anaerobic conditions, since the swimming phenotypes of hypermotile mutants affected in flagella synthesis (*gacA*, *algU*, *kinB*, *sadB*) [22] and flagella rotation (*wspR*) [24] are similar under both conditions. This is not the case for chemotaxis, since different mutants have different phenotypes under aerobic and anaerobic conditions, indicating differences in chemotactic behavior.

Conversely to other pseudomonads, which harbor one or two chemotaxis apparatus, *P*. *fluorescens* Subgroup I strains, such as F113 and NFM421 harbor three, named Che1, Che2 and Che3 [4]. Che1 is the canonical chemotaxis system present in all pseudomonads. Similarly to other species, in F113 the genes encoding this system are located in two widely spaced gene clusters (PsF113_1586–1594 and PsF113_4455–4456). This system is absolutely required for chemotaxis in *P*. *aeruginosa* [29] and we have observed the same for F113, both under aerobic and anaerobic conditions, indicating that in F113, the Che1 system is also the most important chemotaxis system. The F113 Che3 system is encoded by genes located in a single gene cluster (PsF113_3554–3563) that contains two genes encoding MCPs. This gene cluster shows synthenic organization with the Che2 system in *P*. *aeruginosa*, although gene homology is limited. In *P*. *aeruginosa* it has been shown that this system is not essential for chemotactic motility, but it is required for optimal chemotaxis [29]. We have observed a similar phenotype for a mutant affected in the *cheA3* gene under aerobic conditions. However, the F113 *cheA3* mutant did not show chemotactic motility under anaerobic, denitrifying conditions, suggesting that the Che3 system is absolutely required for chemotaxis under anaerobic conditions. To our knowledge, the implication of chemotaxis systems in *P*. *aeruginosa* or other pseudomonads has not been tested under denitrification conditions. It is therefore possible that the *P*. *aeruginosa* Che2 system is also implicated in chemotaxis under denitrification conditions. In this sense, it has been shown that McpB, a methyl accepting protein encoded within the Che2 gene cluster, contributes to aerotaxis in *P*. *aeruginosa* [32, 33]. The F113 Che2 system is encoded by a gene cluster (PsF113_2284–2292) paralogous to the F113 Che3 cluster. Mutation of the *cheA2* gene has shown that this system is not essential for chemotactic motility under aerobic or anaerobic conditions. However, similarly to Che2 in *P*. *aeruginosa* [34], optimal chemotaxis requires this system, both under aerobic and anaerobic conditions. Anyway, the lower degree of halo reduction, indicates that the Che2 system is less important than the Che3 system in F113 for chemotaxis. The results presented here show that flagella synthesis and rotation is regulated in a similar way under aerobic and anaerobic conditions. On the other hand, chemotactic signal transduction is different under aerobic and anaerobic conditions. We have also shown that the three systems are functional and independent. In *P*. *aeruginosa*, similar conclusions were reached, since Che1 and Che2 proteins did not form hybrid complexes [34].

Chemotaxis has been shown to play an important role for rhizosphere competitive colonization [21]. In *P*. *fluorescens* Pf01 it has been shown that mutation in MCPs which detect aminoacids [35] and organic acids [36] present in root exudates are affected in rhizosphere colonization. In order to test the relative importance of each chemotactic system in F113, we have performed competitive colonization assays. The results clearly show that the non-chemotactic mutant affected in *cheA1* was displaced from the rhizosphere by its isogenic wild-type strain, confirming the importance of chemotaxis for rhizosphere colonization. Both *cheA2* and *cheA3* mutants also showed a defect, although minor for competitive colonization. The least affected mutant was *cheA2*, in accordance with its proposed minor role in chemotaxis.

## Material and Methods

### Bacterial strains, plasmids and growth conditions

The strains and plasmids used in this study are described in S1 Table. For growing *P*. *fluorescens* F113 strains under aerobic conditions we have used either SA medium [37] or LB medium [38] with shaking overnight at 28°C. When growing under anaerobic conditions, SA or LB media were supplemented with nitrate (KNO_**3**_) or nitrite (NaNO_2_) as the final electron acceptor in airtight tubes that were flushed with argon after inoculation. Growth was monitored by determining optical density of cultures (OD_600_) at different time intervals using a biophotometer (Eppendorf BioPhotometer D30). Anaerobic conditions for growth on solid media were provided by anaerobic jars and systems (Oxoid). *Escherichia coli* strains were grown overnight in Luria- Bertani (LB) medium with shaking at 37°C. Solid growth media contained 1.5% (w/v) agar. The following antibiotics were used, when required, at the indicated concentrations: rifampicin (Rif), 100 μg/mL; ampicillin (Amp), 100 μg/mL; tetracycline (Tet), 10 μg/mL for *E*. *coli* or 70 μg/mL for *P*. *fluorescens* F113; kanamycin (Km), 25 μg/mL for *E*. *coli* or 50 μg/mL for *P*. *fluorescens* F113; and gentamicin (Gm), 10 μg/mL for *E*. *coli* or 3 μg/mL for *P*. *fluorescens* F113 and chloramphenicol 30 μg/mL for *E*. *coli*. All growth experiments were performed 4 times in triplicate.

### Construction of mutants

Insertional mutagenesis has been used to generate mutants by single homologous recombination. Amplified internal fragments from the different genes to be interrupted were cloned into the kanamycin-resistant plasmid pK19mobsac [39] (see S2 Table) and introduced into the wild-type F113 strain by triparental mating using pRK600 as the helper plasmid [40]. Mutants resulting from the single homologous recombination were checked by Southern blotting using probes from the interrupted genes, and by PCR using primers designed from the genes and the pK19mobsac plasmid sequences. Mutant complementation analysis was done by isolating cosmids containing the genes from the F113 gene bank of F113. The selected cosmids were introduced in the corresponding mutant by triparental mating using pRK600 as the helper plasmid.

### Motility assays

Swimming motility was tested on SA and LB medium with 0.3% (w/v) of purified agar. The same media supplemented with 20 mM nitrate (KNO_3_) and 10 mM (NaNO_2_) nitrite were used to test swimming under anaerobic conditions. Plates were inoculated with bacteria from an overnight culture using a sterile toothpick and incubated at 28°C. Swimming haloes were measured after 18 and 24 h of inoculation under aerobic conditions, and after 48 and 72 h of inoculation under anaerobic conditions. Every assay was done in triplicate at least three times.

### Rhizosphere competitive colonization assays

A root-tip assay test [5] was used. Alfalfa seeds (*Medicago sativa* var. Resis) were sterilized in 70% ethanol for 2 min and in diluted bleach (1:5 v/v) for 15 min and rinsed thoroughly with sterile distilled water. Seeds were germinated at 4°C for 16 h followed by incubation in darkness at 28°C for 24 hours. Germinated alfalfa seeds were sown in Leonard jar gnotobiotic systems using Perlite as solid substrate and 8 mM KNO_3_ supplemented FP [41] as the mineral solution. After 2 days, alfalfa seedlings were inoculated with ∼10^8^ cells of the appropriate strains (10^8^ cells/ml = 0.0138 optical density at 600 nm). For the competitive colonization assays, the tested strain and the competitor were inoculated at a 1:1 ratio. Plants were maintained for 2 weeks in controlled conditions (16 h of light at 25°C and 8 h of dark at 18°C). Bacteria were recovered from the root tip (last centimeter of the main root) by vortexing for 2 min in 5 ml of SA and appropriate dilutions were plated in SA supplemented with selective antibiotics and after 48 hours of incubation at 28°C, colonies were counted. Colonization assays were done three times in triplicate with 20 plants per replica.

### Statistical analysis

SPSS17.0 software (IBM) or Sigma Plot 12.0 software (Microsoft) was used for statistical analysis. The data were compared using one way analysis of variance (ANOVA) followed by Bonferroni’s multiple comparison test (p<0.05).

## Supporting Information

S1 Fig Flagella visualization. (TIF)

S2 Fig Genetic organization of the three chemotactic systems. (TIF)

S1 Table Bacterial strains and plasmids. (DOCX)

S2 Table Primers used in this study. (DOCX)
