# Supplementary figures and images for: Chemotactic Motility of Pseudomonas fluorescens F113 under Aerobic and Denitrification Conditions
Source: PLoS One. 2015 Jul 10;10(7):e0132242. doi: 10.1371/journal.pone.0132242 (PMC4498747; doi:10.1371/journal.pone.0132242)

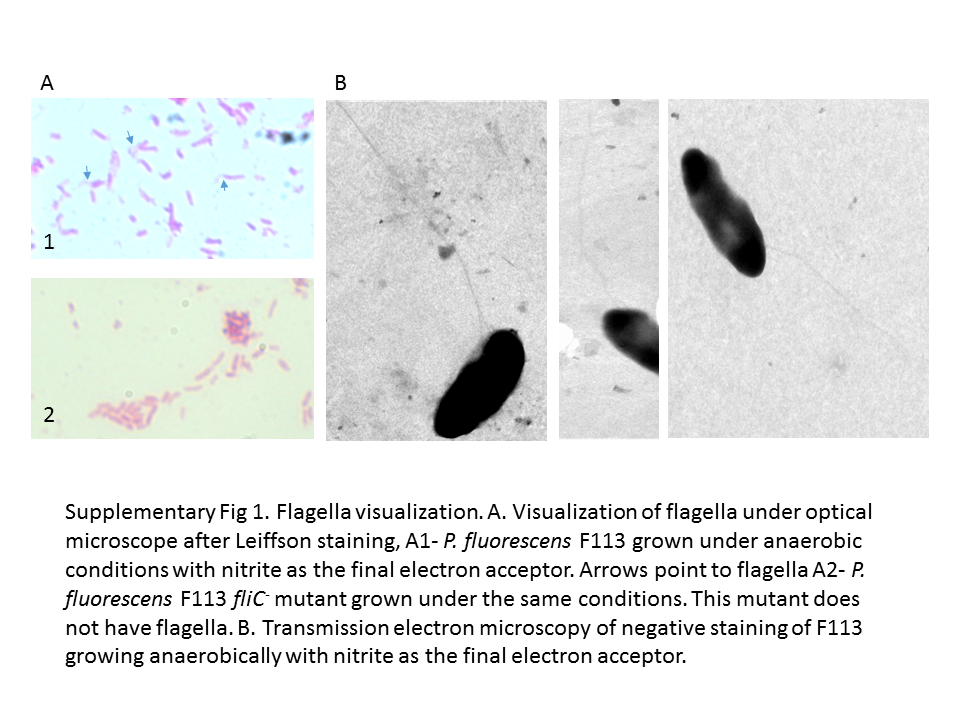

Supplement: S1 Fig — (TIF) [file pone.0132242.s001.tif]

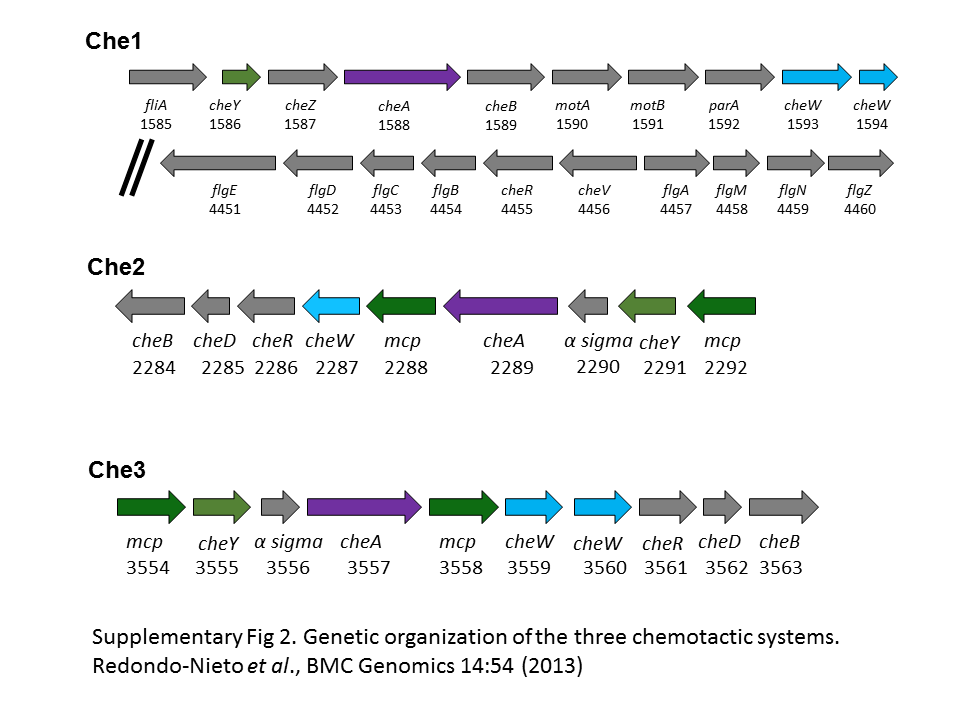

Supplement: S2 Fig — (TIF) [file pone.0132242.s002.tif]
