# Supplementary material for: Chemotactic Motility of Pseudomonas fluorescens F113 under Aerobic and Denitrification Conditions
Source: PLoS One. 2015 Jul 10;10(7):e0132242. doi: 10.1371/journal.pone.0132242 (PMC4498747; doi:10.1371/journal.pone.0132242)
Supplement: S1 Table — (DOCX) [file pone.0132242.s003.docx]

Supplementary Table 1

Bacterial strains and plasmids used in this study

| Strains and plasmids | Description | Reference or source |
| --- | --- | --- |
| Strains |  |  |
| DH5α | *E.coli* cloning strain | Gibco-BRL |
| F113rif | *P. fluorescens* wild-type Rif^R^ | Shanahan *et al.* (1992) |
| F113*cheA1^-^* | F113rif *cheA1^-^* Rif^R^ Km^R^ | This work |
| F113*cheA2^-^* | F113rif *cheA2^-^* Rif^R^ Km^R^ | This work |
| F113*cheA3*^-^ | F113rif *cheA3*^-^ Rif^R^ Km^R^ | This work |
| F113*gacS^-^* | F113rif *gacS^-^* Rif^R^ Km^R^ | Martinez-Granero *et al*. (2006) |
| F113*sadB^-^* | F113rif *sadB^-^* Rif^R^ Km^R^ | Navazo *et al.* (2009) |
| F113*algU^-^* | F113rif *algU^-^* Rif^R^ Km^R^ | Martinez-Granero *et al*. (2012) |
| F113*wspR^-^* | F113rif *wspR^-^* Rif^R^ Spc^R^ | Navazo *et al.* (2009) |
| F113*kinB^-^* | F113rif *kinB^-^* Rif^R^ Km^R^ | Barahona *et al*. (2011) |
| F113p*cheA1* | F113rif *cheA1* pLFAR3*cheA1* Rif^R^ Km^R^ Tc^R^ | This work |
| F113p*cheA2* | F113rif *cheA2* pLFAR3*cheA2* Rif^R^ Km^R^ Tc^R^ | This work |
| F113p*cheA3* | F113rif *cheA3* pLFAR3*cheA3* Rif^R^ Km^R^ Tc^R^ | This work |
|  |  |  |
| Plasmids |  |  |
|  |  |  |
| pGEM-T Easy vector | Cloning vector; Amp^R^ | Promega |
| pRK600 | Helper plasmid, Cm^R^ | Finan *et al.* (1986) |
| pK19mobsacB | pUC18 derivative lacZ mob site sacB, KmR | Schäfer A *et al.* (1994) |
| pBG 2076 | pLAFR3 derivative containing the *cheA1* gen; Tc^r^ | This work |
| pBG 1994 | pLAFR3 derivative containing the *cheA2* gen; Tc^r^ | This work |
| pBG 1988 | pLAFR3 derivative containing the *cheA3* gen; Tc^r^ | This work |

**References**

Shanahan P, O’Sullivan DJ, Simpson P, Glennon JD, O’Gara F (1992) Isolation

of 2,4-diacetylphloroglucinol from a fluorescent pseudomonad and investigation

of physiological parameters influencing its production. Appl Environ Microbiol

58: 353–358.

Martinez-Granero F, Rivilla R, Martín M. (2006). Rhizosphere selection of highly motile phenotypic variants of *Pseudomonas fluorescens* with enhanced competitive colonization ability. Appl Environ Microbiol 72:3429-3434.

Navazo A, Barahona E, Redondo-Nieto M, Martinez-Granero F, Rivilla R, et al. (2009). Three independent signalling pathways repress motility in *Pseudomonas fluorescens* F113. Microb Biotechnol 2: 489–498.

Martínez-Granero F, Navazo A, Barahona E, Redondo-Nieto M, Rivilla R, Martín M. (2012). The Gac-Rsm and SadB signal transduction pathways converge on AlgU to downregulate motility in *Pseudomonas fluorescens*. PLoS One.;7(2):e31765.

Barahona E, Navazo A, Martinez-Granero F, Zea-Bonilla T, Perez-Jimenez

RM, et al. (2011) Pseudomonas fluorescens F113 mutant with enhanced competitive

colonization ability and improved biocontrol activity against fungal root pathogens. Appl Environ Microbiol 77: 5412–5419

Finan TM, Kunkel B, De Vos GF, Signer ER (1986) Second symbiotic megaplasmid in Rhizobium meliloti carrying exopolysaccharide and thiamine

synthesis genes. J Bacteriol 167: 66–72.

Scher FM, Baker R (1982) Effects of Pseudomonas putida and a synthetic iron

chelator on induction of soil suppressiveness to Fusarium wilt pathogens.

Phytopathology 72: 1567–1573.
