# Supplementary material for: Chemotactic Motility of Pseudomonas fluorescens F113 under Aerobic and Denitrification Conditions
Source: PLoS One. 2015 Jul 10;10(7):e0132242. doi: 10.1371/journal.pone.0132242 (PMC4498747; doi:10.1371/journal.pone.0132242)
Supplement: S2 Table — (DOCX) [file pone.0132242.s004.docx]

**Supplementary Table 2**

Primers used in this study

| **NAME** | **PRIMERS SEQUENCE (5´-3´)** | **Tm**  **(ºC)** | **LENGTH of amplicon** |
| --- | --- | --- | --- |
| **CheA1 F** | CAGTATGTTCAGCGAAGTGC | 60 | 608 bp |
| **CheA1 R** | CCTTTGCCATGCAGCTCATC | 62 |  |
| **CheA2 F** | CAGCGATCATATGCTGGAGC | 62 | 681 bp |
| **CheA2 R** | CACCAGGTTGATCAGTTCGT | 60 |  |
| **CheA3 F** | GAGTGCCTTGTTCCTGGAAG | 62 | 709 bp |
| **CheA3 R** | GAATCAGATTACCTTCTGCCG | 62 |  |
| **CheA1*tot* F** | GGAGCAGCCATTAATGAGC | 58 | 2270 bp |
| **CheA1*tot* R** | CCAGAATCAAATACGCCGG | 58 |  |
| **CheA2*tot* F** | GGATATGAGCGTGAGCAT | 58 | 2102 bp |
| **CheA2*tot* R** | GAATTGCTAAAGCGTCAGC | 56 |  |
| **CheA3*tot* F** | CGCCCATGAATCTGGACG | 58 | 2129 bp |
| **CheA3*tot* R** | GAACTATTGAGTCAATTCAGG | 58 |  |
|  |  |  |  |
|  |  |  |  |
